# Association between TyG-related indices and in-hospital acute heart failure in patients with acute myocardial infarction after emergency percutaneous coronary intervention

**DOI:** 10.3389/fendo.2026.1798955

**Published:** 2026-03-06

**Authors:** Rong Feng, Lingling Hu, Ying Zhu, Xun Liang, Tao Tao, Wenjie Li

**Affiliations:** Department of Cardiology, Shehong People’s Hospital, Suining, China

**Keywords:** acute heart failure, acute myocardial infarction, body mass index, emergency percutaneous coronary intervention, triglyceride-glucose index, waist circumference, waist height ratio

## Abstract

**Aim:**

Acute heart failure (AHF) may still occur during hospitalization in some patients with acute myocardial infarction (AMI) after emergency percutaneous coronary intervention (PCI). Triglyceride-glucose (TyG)-related indices are useful markers for evaluating insulin resistance. However, the association between TyG-related indices and in-hospital AHF in AMI patients following emergency PCI remains incompletely understood. The present study aimed to investigate the relationship between TyG-related indices and in-hospital AHF in these patients, with the goal of providing potential clinical evidence for the early identification and management of high-risk individuals.

**Methods:**

This retrospective study enrolled patients with AMI who underwent emergency PCI at our hospital from January 2023 to December 2025. LASSO regression analysis was applied to screen relevant variables. Logistic regression, restricted cubic spline curves and subgroup analysis were performed to evaluate the effects of key variables.

**Results:**

A total of 885 patients were enrolled. Levels of the TyG index, TyG−body mass index (TyG−BMI), TyG−waist−to−height ratio (TyG−WHtR), and TyG−waist circumference (TyG−WC) were significantly higher in patients with AHF. LASSO regression identified 12 risk variables, which were further analyzed using logistic regression and subgroup analyses. Logistic regression demonstrated that the TyG−WHtR was associated with a significantly elevated risk of AHF (OR: 2.29, 95% CI: 1.35–3.91, *P* < 0.01).Receiver operating characteristic (ROC) curve analysis indicated that the TyG−WHtR exhibited a relatively high discriminative performance for AHF, with an area under the curve (AUC) of 0.759 (95% CI: 0.721–0.797). TyG−related indices were stratified and analyzed by quartiles. Results showed that higher quartiles were significantly associated with an increased risk of AHF across all models. Restricted cubic spline (RCS) models revealed linear relationships between TyG, TyG−BMI, TyG−WHtR, TyG−WC and AHF following adjustment for covariates (*P*−overall < 0.05, *P*−nonlinear > 0.05). Subgroup analysis indicated that the TyG−BMI was more strongly associated with incident AHF among patients with the left anterior descending artery (LAD) as the culprit vessel (*P* for interaction < 0.05).

**Conclusions:**

Poor control of TyG-related indices is associated with an increased in-hospital risk of AHF in AMI patients after emergency PCI. Among these indices, the TyG-WHtR may serve as a relatively important and independent predictor.

## Introduction

Acute myocardial infarction (AMI) is a common and leading cause of heart failure (HF) (1). Percutaneous coronary intervention (PCI) represents the first-line therapeutic strategy for patients with AMI (2). However, clinical practice has demonstrated that acute heart failure (AHF) still develops in a proportion of AMI patients following emergency PCI. Without timely intervention and appropriate management, this complication significantly prolongs hospital stay and increases the risk of mortality in affected patients (3). Therefore, identifying predictive biomarkers for AHF risk in this population is critical for the early prevention and optimized management of this condition.

Insulin resistance (IR) is characterized by diminished tissue sensitivity to insulin, resulting in impaired glucose uptake into cells (4). Accumulating evidence has indicated that IR contributes to the development of heart failure (HF) by disrupting cardiomyocyte structure and function (5, 6). The hyperinsulinemic-euglycemic clamp is currently recognized as the gold standard for the diagnosis of IR (7). Nevertheless, its clinical utility is restricted by complicated procedures and high costs. In contrast, the TyG index and its related composite indices have been proposed as reliable surrogate markers of IR, owing to their simplicity in calculation and ready availability in routine clinical practice (8).

Obesity has now become a major public health concern and is closely associated with IR and metabolic disorders in the human body (9, 10). Recent studies have indicated that the TyG index is closely associated with adverse outcomes in patients with HF and can serve as a predictive marker for 30-day and 1-year all-cause mortality risk in this patient population (11). Furthermore, a prospective study involving 7335 participants demonstrated that TyG-Body Mass Index (TyG-BMI), TyG-Waist-to-Height Ratio (TyG-WHtR) and TyG-waist circumference indices (TyG-WC) were significantly associated with the risk of HF during a median follow-up period of 2.97 years (12).

However, no studies have yet investigated the association between the TyG index and its related indices and the risk of in-hospital AHF in patients with AMI who have undergone emergency PCI. This study aimed to characterize and analyze the correlations of the TyG index, TyG-BMI, TyG-WC and TyG-WHtR with the risk of AHF in patients with AMI following emergency PCI.

## Materials and methods

### Data source and study population

**Figure 1:**
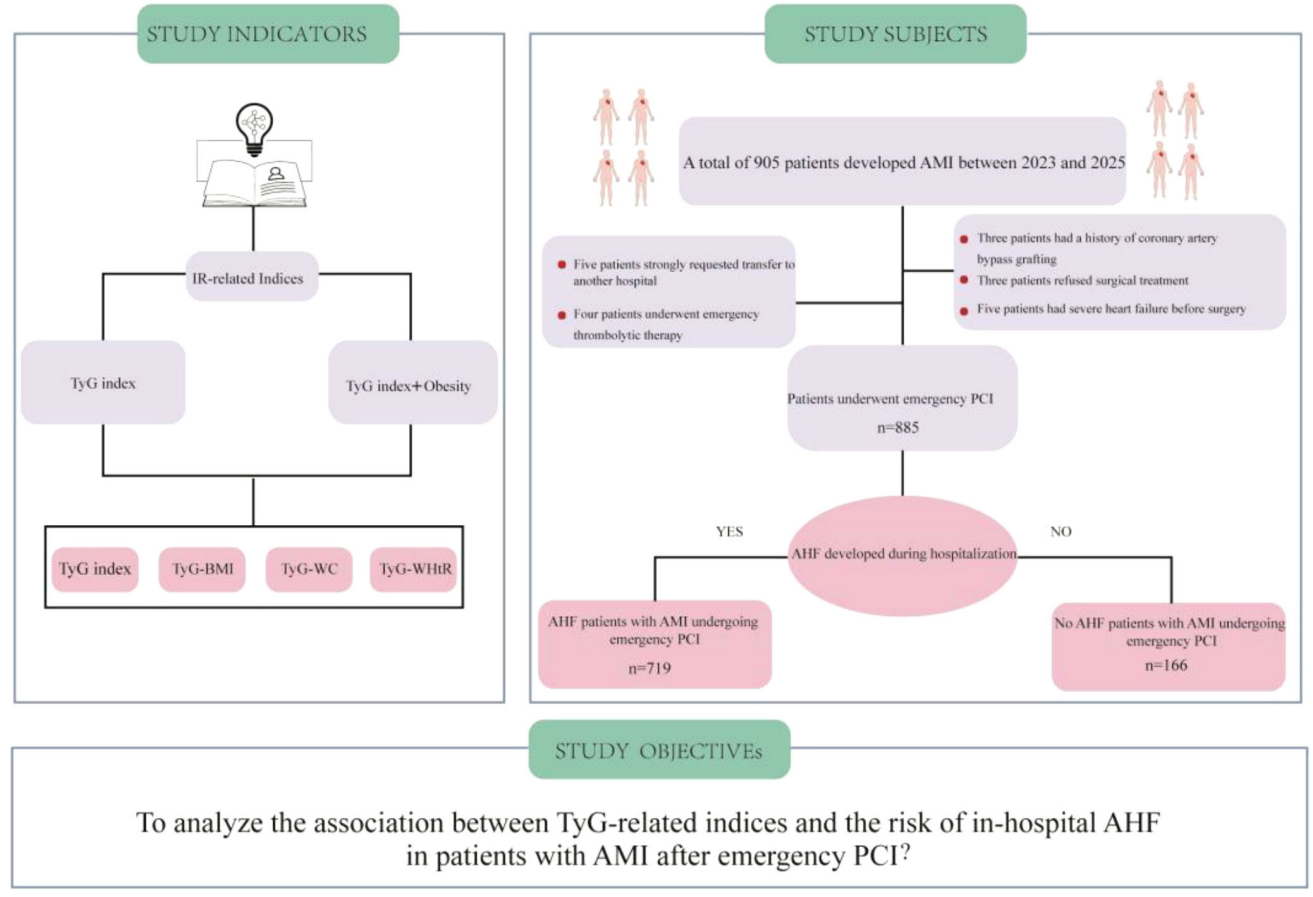
The patient flow chart.

This study retrospectively analyzed patients with AMI who underwent emergency PCI at Shehong People’s Hospital from January 2023 to December 2025. Inclusion criteria were: (1) Patients aged ≥18 years; (2) Patients diagnosed with AMI in accordance with the 2025 American Guidelines for Percutaneous Coronary Intervention and willing to undergo emergency PCI (13); Exclusion criteria included: (1) Patients with a history of coronary artery bypass grafting; (2) Patients with severe HF unable to lie flat; (3) Patients who refused coronary angiography or primary PCI; (4) Patients who had received emergency thrombolytic therapy before surgery; (5) Patients who strongly requested transfer to another hospital. Ultimately, 885 patients who met the inclusion and exclusion criteria were enrolled in the study (**Figure 1**). This study was approved by the Ethics Committee of Shehong People’s Hospital (2025-Shen 51). Given that this was a retrospective study involving identifiable patient identity data and intervention measures, the requirement for informed consent was waived upon application to the hospital.

### Definitions and calculation of related variables

Acute heart failure (AHF) was defined as the rapid onset or acute exacerbation of signs and symptoms of heart failure, accompanied by elevated plasma natriuretic peptide concentrations, a condition that is often life-threatening. Its clinical manifestations comprise a spectrum of signs and symptoms dominated by pulmonary congestion, systemic congestion, and hypoperfusion of tissues and organs. The diagnostic threshold for N-terminal pro-B-type natriuretic peptide (NT-proBNP) was set at ≥300 pg/mL. Diagnosis of AHF was guided by age-stratified thresholds, combined with clinical features including chest tightness, shortness of breath, and signs of pulmonary and systemic congestion: <55 years: NT-proBNP >450 pg/mL; 55–75 years: NT-proBNP >900 pg/mL; >75 years: NT-proBNP >1800 pg/mL (14). NT-proBNP was measured before PCI to exclude patients with pre-existing AHF. Following PCI, NT-proBNP was assayed immediately in patients who developed the aforementioned symptoms during hospitalization to establish a diagnosis of AHF.

Diabetes mellitus was defined as a history of previous diabetes mellitus, or fasting plasma glucose (FPG) ≥7.0 mmol/L after admission, or random plasma glucose ≥11.1 mmol/L, or 2-hour plasma glucose level ≥11.1 mmol/L in a 75-gram oral glucose tolerance test, or glycated hemoglobin ≥6.5% (15).

Smoking history was defined as smoking at least one cigarette per day for a continuous or cumulative period of 6 months or more (16).

Drinking was defined as drinking at least once a week for a continuous or cumulative period of 6 months or more (17).

Fasting blood glucose, blood lipids and other laboratory results were collected from patients immediately upon hospital admission, along with anthropometric parameters including height, body weight and waist circumference. The TyG index, TyG-BMI, TyG-WC and TyG-WHtR were then calculated using the corresponding formulas (18).


$$\text{BMI}=\text{Weight}(\text{kg})/\text{Height}{\left(\text{m}\right)}^{2}$$

WHtR=Weight(kg)/Height(m).

TyG index=Ln[1/2×FPG (mg/dL)×TG (mg/dL)].

TyG−BMI=BMI×TyG index.

TyG−WHtR=WHtR×TyG index.

TyG−WC=WC×TyG index.

### Data collection

All laboratory tests were completed within 30 minutes of emergency admission, using peripheral venous blood specimens, and were performed by the Department of Laboratory Medicine of our hospital. Echocardiography was conducted within 24 hours during hospitalization after PCI and was performed by the Echocardiography Department of our hospital.Clinical information of patients was retrieved from the hospital electronic medical record system, including demographic data, clinical characteristics, laboratory parameters, echocardiographic indices, surgery-related metrics and anthropometric measurements. Demographic data comprised age, gender, heart rate, smoking history and drinking history. Clinical characteristics included a history of hypertension and diabetes mellitus. Oral medications taken by patients involved antiplatelet, antihypertensive and lipid-lowering agents. Laboratory parameters consisted of neutrophil count (Neu), lymphocyte count (Lym), monocyte count (Mon), alanine aminotransferase (ALT), aspartate aminotransferase (AST), total cholesterol (TC), triglyceride (TG), low-density lipoprotein cholesterol (LDL-C), high-density lipoprotein cholesterol (HDL-C), apolipoprotein B to apolipoprotein A1 ratio (ApoB/A1), serum creatinine (Scr), serum uric acid(UA), serum urea, fasting blood glucose (FBG), cardiac troponin I, and NT⁃proBNP. Echocardiographic indices included left ventricular ejection fraction (LVEF) and left ventricular end-diastolic diameter (LVEDD). Anthropometric measurements included height, body weight and WC. Surgery-related metrics involved the type of culprit vessel (right coronary artery [RCA], left anterior descending artery [LAD], left circumflex artery [LCX], left marginal artery [LAM]) and the number of stents implanted.

### Statistical analysis

Statistical analyses were performed on the data using SPSS version 27.0 (IBM Corporation, Chicago, Illinois, USA) and R software version 4.6.7 (R Foundation for Statistical Computing, Vienna, Austria). Continuous variables were expressed as mean ± standard deviation (SD) or median (interquartile range, IQR). Intergroup differences in normally distributed data were analyzed using the t-test or one-way analysis of variance (ANOVA), while those in non-normally distributed data were examined via the Mann-Whitney U test. Categorical variables were presented as counts and percentages, with intergroup comparisons conducted using the chi-square test or Fisher’s exact test. Relevant variables were screened by least absolute shrinkage and selection operator (LASSO) regression for subsequent inclusion in logistic regression analyses. Logistic regression models were applied to estimate the odds ratios (ORs) and 95% confidence intervals (CIs) for the association between changes in TyG-related indices and the incidence of AHF, and four multivariable models were constructed accordingly. Restricted cubic spline (RCS) regression was used to assess the relationship between TyG-related indices and AHF. Receiver operating characteristic (ROC) curves were generated to calculate the corresponding area under the curve (AUC) and the optimal cut-off values for each variable. Continuous variables were converted into categorical variables based on these optimal cut-off values for subgroup analyses, which were conducted to further evaluate the association between TyG-related indices and AHF; forest plots were plotted for visualization of the results. A two-tailed *P* value < 0.05 was considered statistically significant.

## Results

### Baseline characteristics

A total of 885 patients were enrolled in the study, with a mean age of 62.5 years; 18.8% of them were diagnosed with AHF, and 52% of the total study population were female. The results showed that the TyG index, TyG-BMI, TyG-WHtR, and TyG-WC in patients with AHF were significantly higher than those in subjects without AHF (P < 0.01). AHF was more likely to occur in female patients, older individuals, and those with lower HDL-C levels, higher levels of urea, Scr, UA, BMI, WC, WHtR, FBG, TC, TG, LDL-C, and ApoB/A1, as well as higher cardiac troponin I levels. Additionally, AHF was more prevalent in patients whose culprit vessel was the LAD and those with a larger number of implanted stents (**Table 1**).

**Table 1:** Baseline characteristics of patients with and without acute heart failure.

Characteristic	Overall N = 885	Non-AHF N = 719	AHF N = 166	p-value
Age (years), Median (IQR)	63.00 (57.00, 69.00)	62.00 (56.00, 68.00)	64.00 (59.00, 71.00)	<0.001
BMI (kg/m^2^), Median (IQR)	23.57 (22.30, 25.34)	23.44 (22.15, 25.34)	23.98 (22.66, 25.63)	0.013
Height(m), Median (IQR)	1.67 (1.63, 1.72)	1.67 (1.63, 1.72)	1.68 (1.64, 1.73)	0.27
WHtR, Median (IQR)	0.40 (0.37, 0.43)	0.40 (0.37, 0.43)	0.41 (0.38, 0.43)	0.02
Urea (mg/dL), Median (IQR)	5.70 (4.70, 6.70)	5.70 (4.70, 6.67)	6.10 (4.80, 6.90)	0.035
Scr (mg/dL), Median (IQR)	0.77 (0.66, 0.87)	0.77 (0.66, 0.86)	0.78 (0.71, 0.89)	0.005
UA(mg/dL), Median (IQR)	5.49 (4.38, 6.38)	5.43 (4.30, 6.30)	5.78 (4.88, 6.62)	0.003
Neu, Median (IQR)	4.04 (3.07, 5.17)	4.04 (3.06, 5.17)	4.07 (3.40, 5.11)	0.21
Lym, Median (IQR)	1.73 (1.38, 2.16)	1.73 (1.36, 2.16)	1.75 (1.44, 2.15)	0.75
Mon, Median (IQR)	0.48 (0.39,0.57)	0.48 (0.38, 0.57)	0.49 (0.42, 0.56)	0.17
WC (cm), Median (IQR)	95.00 (90.00, 101.00)	95.00 (89.00, 100.00)	96.00 (91.00, 102.00)	0.004
FPG (mg/dl), Median (IQR)	100.80 (88.20, 122.40)	99.00 (87.20, 121.80)	107.50 (91.80, 125.40)	0.003
TyG-BMI, Median (IQR)	205.41 (193.85, 222.72)	204.40 (193.15, 221.46)	209.76 (198.49, 229.73)	0.001
TyG index, Median (IQR)	8.74 (8.55, 8.90)	8.74 (8.53, 8.89)	8.78 (8.65, 8.98)	0.006
TyG-WHtR, Median (IQR)	3.47 (3.23, 3.76)	3.44 (3.22, 3.74)	3.56 (3.32, 3.83)	0.001
TyG-WC, Median (IQR)	834.20 (779.45, 886.00)	831.29 (772.20, 882.74)	848.06 (811.68, 901.25)	<0.001
TC (mg/dl), Median (IQR)	175.29 (155.28, 220.65)	173.74 (153.52, 223.95)	192.00 (167.77, 211.97)	0.037
TG (mg/dl), Median (IQR)	124.40 (100.96, 145.99)	123.43 (99.17, 145.96)	127.41 (108.02, 148.18)	0.029
HDL-C (mg/dl), Median (IQR)	50.55 (42.54, 62.48)	51.82 (43.31, 63.03)	45.57 (38.28, 55.32)	<0.001
LDL-C (mg/dl), Median (IQR)	93.42 (78.89, 105.57)	90.87 (76.18, 104.41)	98.92 (92.31, 108.01)	<0.001
ApoB/A1, Median (IQR)	1.88 (1.51, 2.43)	1.86 (1.48, 2.39)	2.03 (1.59, 2.53)	0.031
AST (U/L), Median (IQR)	20.00 (18.00, 25.00)	20.00 (18.00, 25.00)	21.00 (17.00, 26.00)	0.97
ALT (U/L), Median (IQR)	21.00 (15.00, 28.00)	21.00 (16.00, 28.00)	21.50 (15.00, 31.00)	0.5
LVEDD (cm), Median (IQR)	47.00 (45.00, 50.00)	47.00 (45.00, 50.00)	47.50 (44.00, 49.00)	0.67
LVEF (%), Median (IQR)	62.00 (58.00, 66.00)	62.00 (58.00, 66.00)	62.00 (58.00, 65.00)	0.24
Cardiac troponin I(ng/l), Median (IQR)	14.40 (13.20, 16.60)	14.40 (13.10, 16.00)	15.80 (14.40, 20.70)	<0.001
Heart, Median (IQR)	77.00 (68.00, 85.00)	77.00 (69.00, 85.00)	78.00 (68.00, 85.00)	0.89
Gender, n (%)				0.017
Female	385 (44)	299 (42)	86 (52)	
Male	500 (56)	420 (58)	80 (48)	
ACEI/ARB/ARNI, n (%)	745 (84)	604 (84)	141 (85)	0.77
Statins, n (%)	880 (99)	715 (99)	165 (99)	1.00
Beta blocker, n (%)	778 (88)	632 (88)	146 (88)	0.99
Antiplatelet agent, n (%)	885 (100)	719 (100)	166 (100)	1.00
Hypertension, n (%)	394 (45)	303 (42)	91 (55)	0.003
Smoking, n (%)	386 (44)	313 (44)	73 (44)	0.92
Drinking, n (%)	180 (20)	149 (21)	31 (19)	0.55
RCA, n (%)	391 (44)	326 (45)	65 (39)	0.15
LAD, n (%)	301 (34)	227 (32)	74 (45)	0.001
LCX, n (%)	189 (21)	162 (23)	27 (16)	0.076
LAM, n (%)	5 (0.6)	4 (0.6)	1 (0.6)	1.00
Nt-proBNP, n (%)	558 (63)	447 (62)	111 (67)	0.26
D-dimer, n (%)	405 (46)	325 (45)	80 (48)	0.49
Number of stents, n (%)				
≤1	333 (38)	251 (35)	82 (49)	<0.001
>1	552 (62)	468 (65)	84 (51)	

AHF, acute heart failure; TyG, triglyceride–glucose; BMI, Body mass index; WHtR, waist height ratio; WC, waist circumference indices; Scr, serum creatinine; UA, uric acid; Neu, neutrophil count; Lym, lymphocyte count; Mon, monocyte count; FBG, fasting blood glucose; TC, total cholesterol; TG, triglyceride; HDL-C, high-density lipoprotein cholesterol; LDL-C, low-density lipoprotein cholesterol; ApoB/A1, apolipoprotein B to apolipoprotein A1 ratio; LVEF, left ventricular ejection fraction; LVEDD, left ventricular end-diastolic diameter; ACEI/ARB/ARNI, angiotensin-converting enzyme inhibitors/angiotensin receptor blockers/angiotensin receptor-neprilysin inhibitors; RCA, right coronary artery; LAD, left anterior descending artery; LCX, left circumflex artery; LAM, left marginal artery.

### Clinical outcome

**Figure 2:**
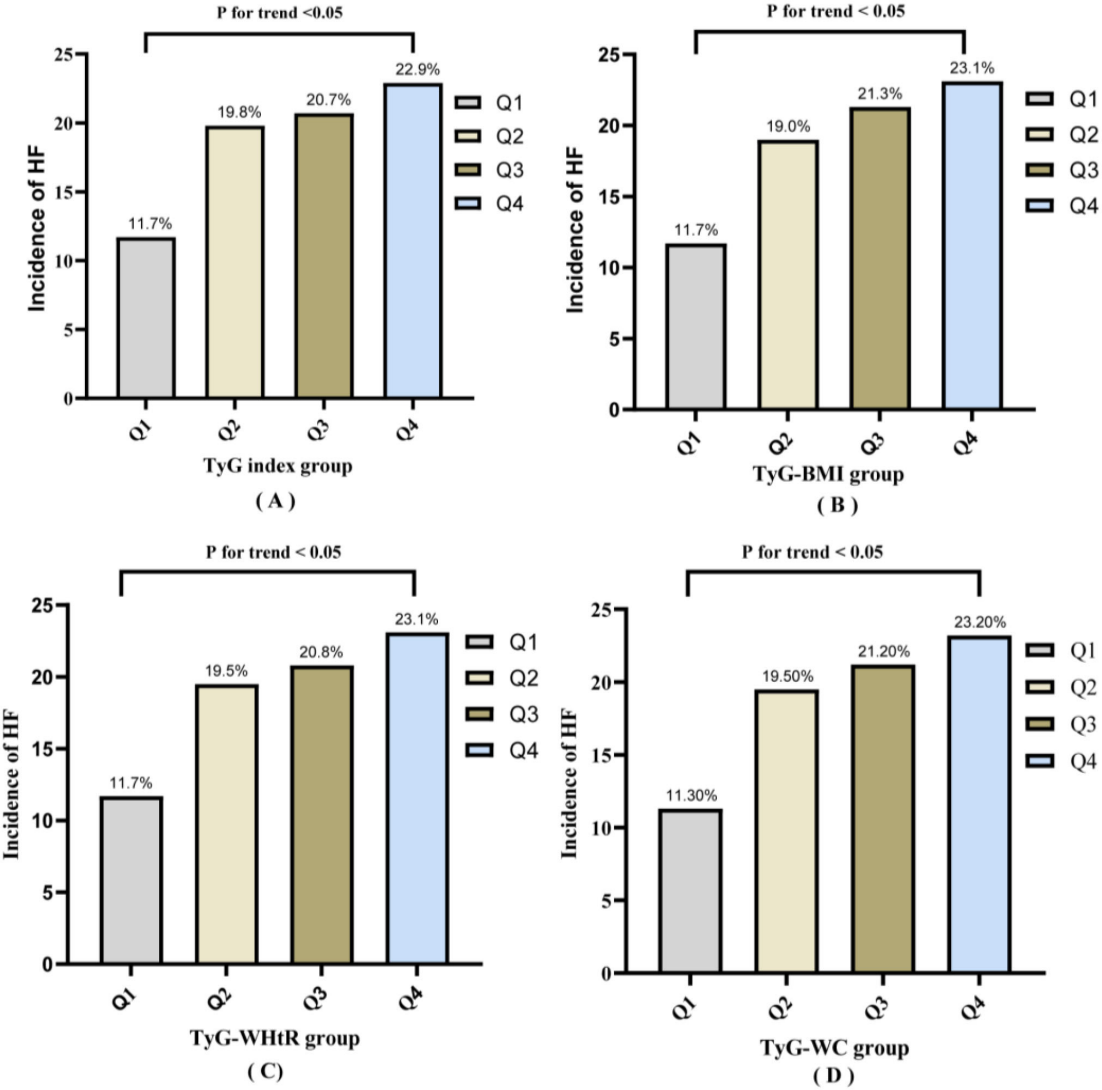
Incidence of AHF stratified by quartiles of the TyG index, TyG-WC, TyG-WHtR, and TyG-BMI. **(A)** Incidence of AHF by quartiles of the TyG index; **(B)** Incidence of AHF by quartiles of the TyG-BMI index; **(C)** Incidence of AHF by quartiles of the TyG-WHtR index; **(D)** Incidence of AHF by quartiles of the TyG-WC index.

As shown in **Figure 2**, patients were stratified by quartiles of the TyG index, TyG-BMI, TyG-WHtR and TyG-WC, with a significant increase in the risk of AHF observed as quartile categories rose (all *P* for rend < 0.05). To explore the potential effects of the TyG index and its related indicators at different levels, patients were stratified into four groups according to the quartile values of the TyG index, TyG-WC, TyG-WHtR and TyG-BMI, respectively. For descriptive convenience, these groups were designated as the first (Q1), second (Q2), third (Q3) and fourth (Q4) quartiles, with the Q1 group serving as the reference category. Among the four indicators, the TyG index was associated with the mildest elevation in AHF risk: the incidence of AHF was 11.7% in the Q1, 19.8% in the Q2, 20.70% in the Q3, and 22.90% in the Q4. In contrast, TyG-WC exhibited the most pronounced increase in AHF risk, with the incidence of AHF being 11.3% in the Q1 group, 19.5% in the Q2 group, 21.20% in the Q3 group, and 23.20% in the Q4 group.

### Screening of relevant variables

**Figure 3:**
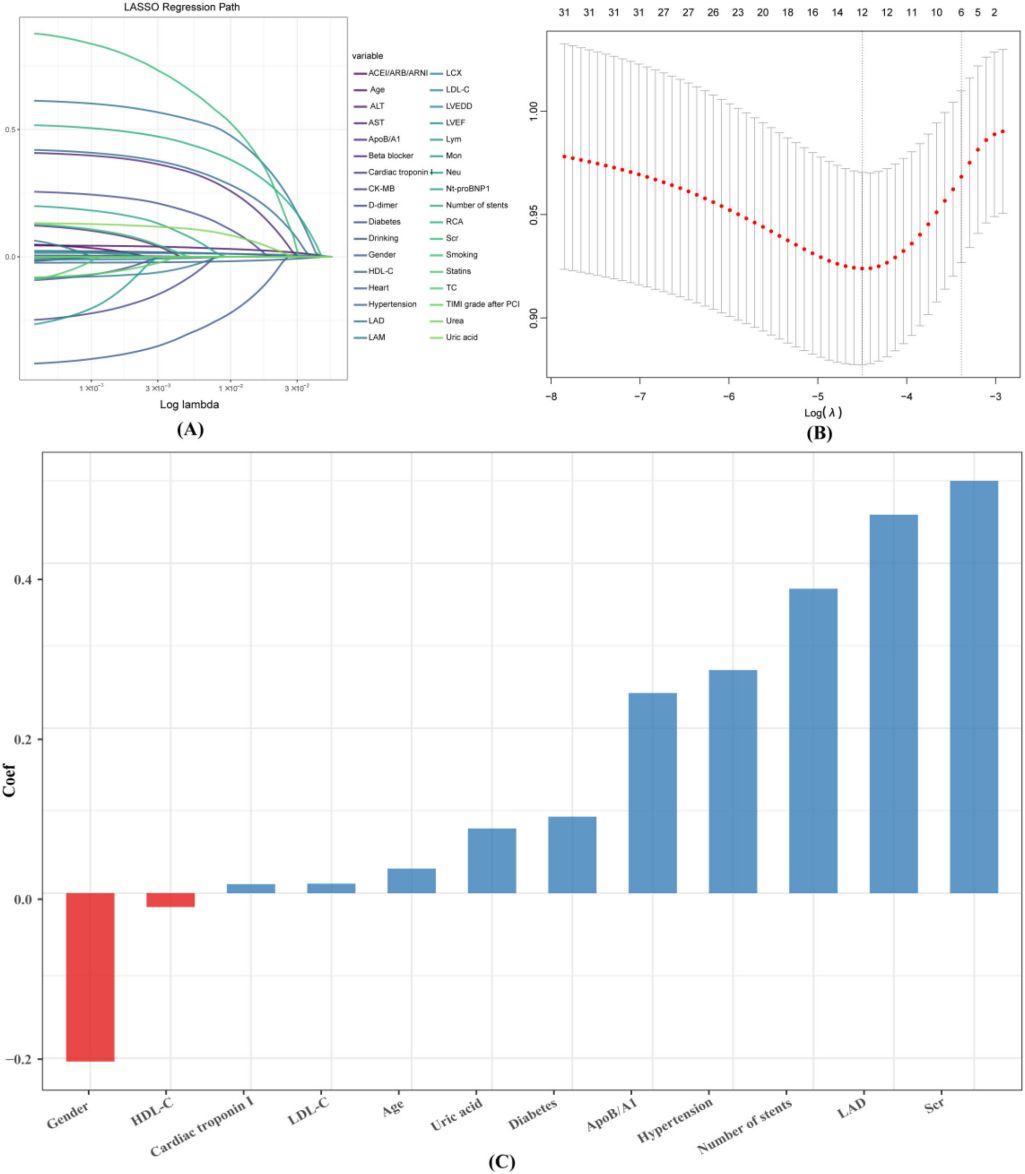
Variable selection via the LASSO logistic regression model. **(A)** Distribution of LASSO coefficients for the 34 features. **(B)** Selection of the optimal λ (lambda) parameter in the LASSO model using ten-fold cross-validation based on the minimum standard error. **(C)** Ranking of the 12 screened feature variables by their coefficient values.

To address and mitigate multicollinearity among metabolic and lipid-related variables, this study employed LASSO regression for variable selection. A key advantage of LASSO regression is its ability to automatically identify predictive variables from high-dimensional datasets with multicollinearity, thereby reducing overfitting. Variables with non-zero coefficients identified by LASSO were subsequently included in conventional multivariate logistic regression. This step was designed to derive interpretable regression coefficients (odds ratios, ORs) and corresponding 95% confidence intervals (CIs), facilitating clinical interpretation and practical application. The LASSO method combined with ten-fold cross-validation was applied to screen the key variables associated with the risk of AHF. The optimal λ (lambda) value, corresponding to the distance of the minimum standard error, was identified as 0.01109. At this threshold, the model selected 12 feature variables: age, gender, hypertension, diabetes mellitus, LDL-C, HDL-C, ApoB/A1, Scr, UA, cardiac troponin I, LAD, and the number of stents. The importance ranking of these 12 feature variables is presented in **Figure 3**.

### Stratification between the TyG index, TyG-BMI, TyG-WHtR, TyG-WC and AHF

12 feature variables identified by LASSO regression were incorporated into four logistic proportional hazard models to investigate the associations between the TyG index, its derived composite indices, and AHF (**Table 2**). Statistically significant positive associations were observed between the TyG index, TyG-BMI, TyG-WC, TyG-WHtR and AHF across all models (*P* < 0.05). After adjustment for confounding factors in Model 4, comparison of the Q4 with the Q1 yielded the following adjusted OR: 2.04 (95% CI, 1.18-3.58) for the TyG index, 2.13 (95% CI, 1.24-3.74) for TyG-BMI,1.99 (95% CI, 1.14-3.54) for TyG-WC, and 2.29 (95% CI, 1.32-4.04) for TyG-WHtR. Tests for trend yielded statistically significant results in all models (*P* < 0.05).

**Table 2:** Associations of the TyG-related indices with AHF to different models.

Characteristic	Model 1		Model 2		Model 3		Model 4	
	OR (95%CI)	*P* value	OR (95%CI)	*P* value	OR (95%CI)	*P* value	OR (95%CI)	*P* value
TyG index	2.56(1.42,4.64)	<0.01	2.58(1.43,4.72)	<0.01	2.29(1.22,4.11)	<0.01	2.21(1.19,4.14)	<0.01
Quartile								
Q1	1 (Reference)		1 (Reference)		1 (Reference)		1 (Reference)	
Q2	1.87(1.11,3.17)	<0.05	2.10(1.24,3.62)	<0.05	2.14(1.24,3.75)	<0.05	2.00(1.15,3.53)	<0.05
Q3	1.98(1.75,3.38)	<0.01	2.20(1.30,3.78)	<0.01	2.37(1.38,4.15)	<0.01	2.26(1.31,3.99)	<0.01
Q4	2.26(1.35,3.78)	<0.01	2.32(1.38,3.96)	<0.01	2.06(1.20,3.58)	<0.01	2.04(1.18,3.58)	<0.01
P for trend	<0.01		<0.01		<0.05		<0.05	
TyG-BMI	1.01(1.00,1.02)	<0.01	1.02(1.01,1.02)	<0.01	1.01(1.00,1.02)	<0.01	1.01(1.00,1.02)	<0.01
Quartile								
Q1	1 (Reference)		1 (Reference)		1 (Reference)		1 (Reference)	
Q2	1.77(1.04,3.00)	<0.05	1.72(1.01,2.97)	<0.05	1.58(0.92,2.77)	<0.05	1.51(0.87,2.67)	<0.05
Q3	2.04(1.21,3.43)	<0.01	2.05(1.22,3.53)	<0.01	2.04(1.19,3.55)	<0.01	2.10(1.22,3.69)	<0.01
Q4	2.26(1.35,3.79)	<0.01	2.52(1.50,4.34)	<0.01	2.23(1.30,3.87)	<0.01	2.13(1.24,3.74)	<0.01
P for trend	<0.01		<0.01		<0.01		<0.01	
TyG-WC	1.01(1.00,1.01)	<0.01	1.01(1.00,1.01)	<0.01	1.01(1.00,1.01)	<0.01	1.01(1.00,1.01)	<0.01
Quartile								
Q1	1 (Reference)		1 (Reference)		1 (Reference)		1 (Reference)	
Q2	1.90(1.12,3.24)	<0.05	2.00(1.17,3.49)	<0.05	1.54(0.88,2.76)	<0.05	1.42(0.80,2.56)	<0.05
Q3	2.12(1.25,3.58)	<0.01	2.31(1.36,4.01)	<0.01	1.92(1.10,3.39)	<0.01	1.82(1.04,3.24)	<0.01
Q4	2.38(1.41,4.00)	<0.01	2.52(1.49,4.36)	<0.01	2.21(1.27,3.92)	<0.01	1.99(1.14,3.54)	<0.01
P for trend	<0.01		<0.01		<0.01		<0.01	
TyG-WHtR	2.18(1.35,3.54)	<0.01	2.79(1.67,4.68)	<0.01	2.40(1.43,4.08)	<0.01	2.29(1.35,3.91)	<0.01
Quartile								
Q1	1 (Reference)		1 (Reference)		1 (Reference)		1 (Reference)	
Q2	1.82(1.08,3.12)	<0.05	1.69(0.99,2.91)	<0.05	1.64(0.95,2.86)	<0.05	1.59(0.92,2.80)	<0.05
Q3	1.98(1.18,3.37)	<0.01	2.04(1.21,3.50)	<0.01	2.02(1.18,3.52)	<0.01	1.96(1.14,3.44)	<0.01
Q4	2.26(1.36,3.83)	<0.01	2.67(1.59,4.65)	<0.01	2.37(1.39,4.16)	<0.01	2.29(1.32,4.04)	<0.01
P for trend	<0.01		<0.01		<0.01		<0.01	

OR, odds ratio; CI, confidence interval.

Model 1: Unadjusted model.

Model 2: Adjusted for age, gender, hypertension, diabetes mellitus.

Model 3: Adjusted for variables included in Model 2+ LDL-C, HDL-C, ApoB/A1, Scr, UA, cardiac troponin I.

Model 4: Adjusted for variables included in Model 3+ LAD, and the number of stents.

### The regression model for investigating the associations between the lated indices and AHF

**Figure 4:**
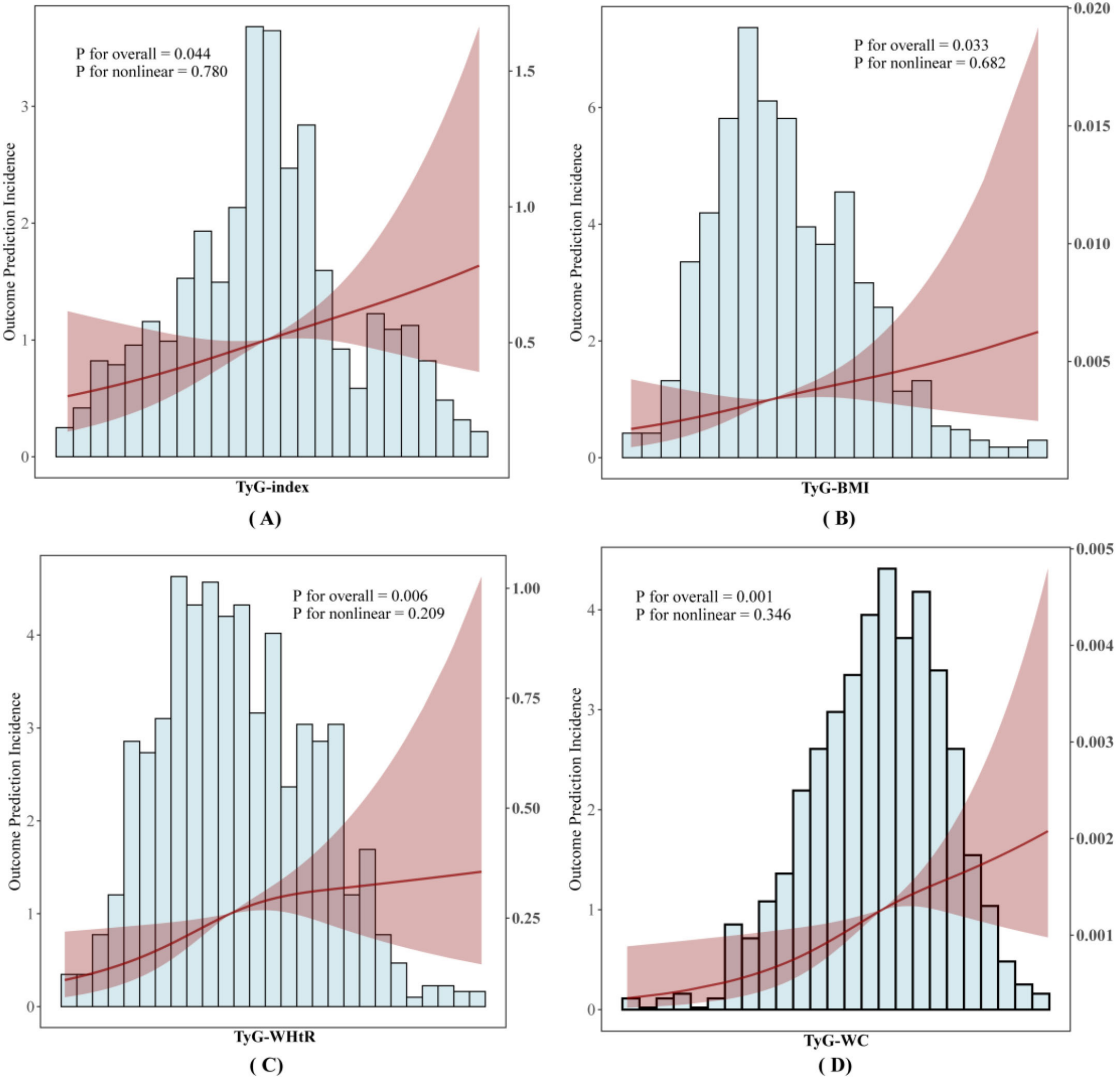
RCS analysis of the association between TyG-related indices and outcome incidence of AHF. **(A)** Association between TyG-index and AHF outcome incidence; **(B)** Association between TyG-BMI and AHF outcome prediction incidence; **(C)** Association between TyG-WHtR and AHF outcome prediction incidence; **(D)** Association between TyG-WC and AHF outcome prediction incidence.

In **Figure 4**, RCS analysis was used to model and visualize the associations of TyG index, TyG-BMI, TyG-WC, and TyG-WHtR with the risk of AHF occurrence. After adjustment for covariates including age, gender, hypertension, diabetes, LDL-C, HDL-C, ApoB/A1, Scr, UA, cardiac troponin I, LAD, and the number of stents, linear associations were observed between each of the TyG index, TyG-BMI, TyG-WC, and TyG-WHtR indices and AHF (*P*-overall < 0.001, *P*-nonlinear > 0.05).

### ROC curves for patients with AHF

**Figure 5:**
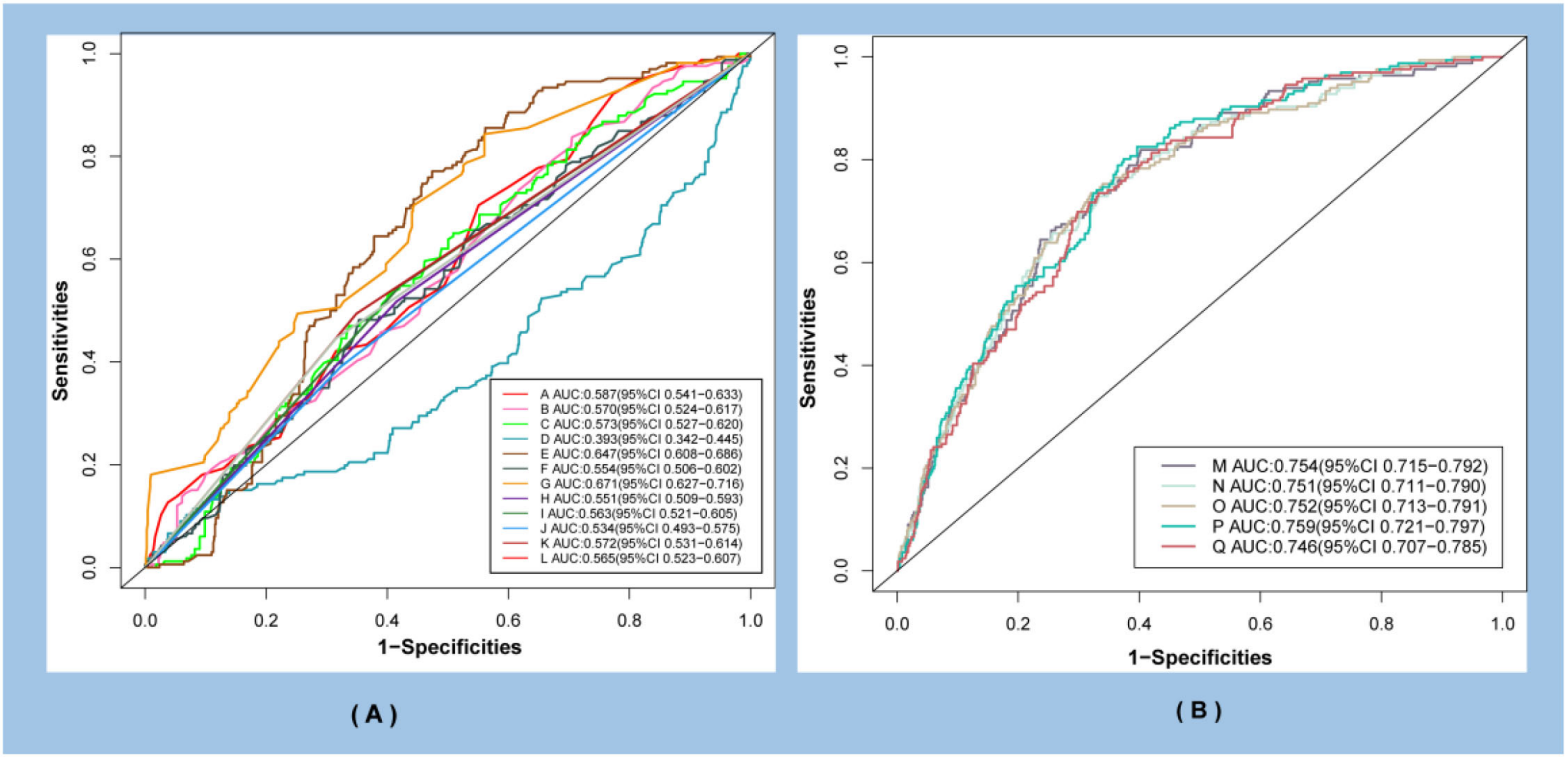
Analysis of the predictive value of ROC curves for AHF after PCI. **(A)** presents the ROC curves for the 12 risk variables selected by LASSO regression. **(B)** presents the ROC curves for TyG-related indices combined with the 12 aforementioned risk variables. A, Age; B, Scr; C, Uric acid; D, HDL-C; E, HDL-C; F, HDL-C; G, Cardiac troponin I; H, Gender; I, Hypertension; J, Diabetes; K, Number of stents; L, LAD; Q, Age, Scr, Uric acid, HDL-C, HDL-C, HDL-C, Cardiac troponin I, Gender, Hypertension, Diabetes, Number of stents; L, LAD; M, TyG index+Q; N, TyG-BMI+Q; O, TyG-WC +Q; P, TyG-WHtR+Q.

We further analyzed the predictive value of seven continuous variables (age, Scr, UA, HDL-C, LDL-C, Apo B/A1, and cardiac troponin I) screened by LASSO regression and five categorical variables (gender, hypertension, diabetes, LAD, and number of stents) for the occurrence of AHF. The predictive performance of TyG−related indices combined with these 12 variables was also evaluated. As shown by the ROC curves in **Figure 5**, TyG-WHtR exhibited the highest predictive capacity for AHF onset compared with other variables (AUC: 0.759, 95% CI: 0.721–0.797). In addition, the Youden index and optimal cutoff value were calculated for the seven continuous variables (**Table 3**). These continuous variables were subsequently converted into categorical variables based on their respective optimal cutoffs. For ease of documentation, variables were labeled sequentially with letters from A to Q.

**Table 3:** Youden’s indices and optimal cut-off values of the seven continuous variables.

Variables	Age	Scr	Uric acid	HDL-C	LDL-C	ApoB/A1	Cardiac troponin I
Area under the curve	0.587	0.570	0.573	0.393	0.647	0.554	0.691
Youden indices	0.170	0.165	0.183	0.127	0.179	0.191	0.169
Optimal cut-off values	60.5	0.665	5.37	55.82	91.5	2.12	13.35

### Risk stratification of TyG index, TyG-BMI, TyG-WHtR and TyG-WC in association with AHF

**Figure 6:**
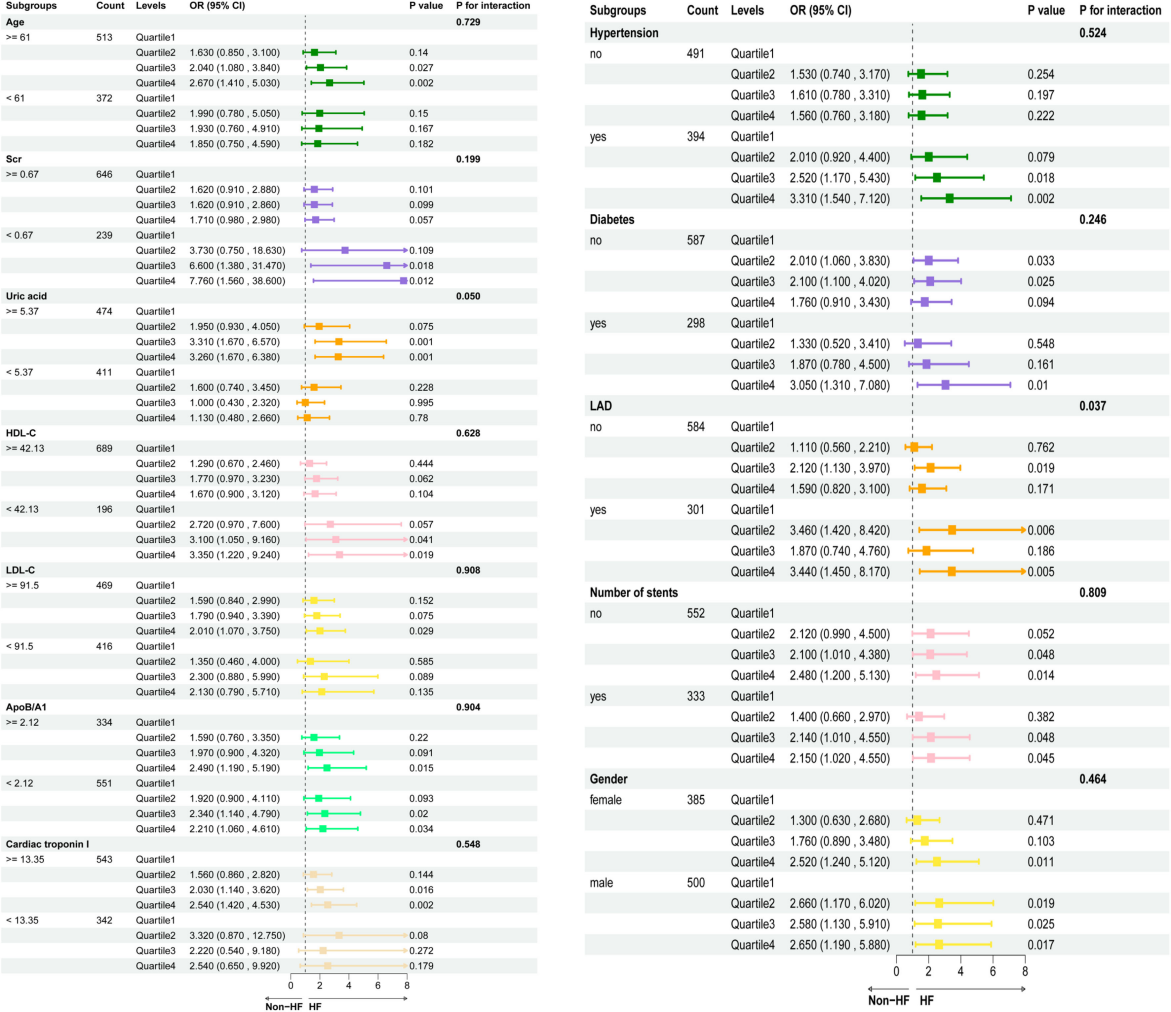
The results of subgroup analyses for the association between the TyG-BMI and AHF.

Continuous variables were converted into categorical variables according to these optimal cut-off values; combined with five categorical variables (gender, hypertension, diabetes mellitus, LAD, and the number of stents), we further investigated the associations of TyG index, TyG-BMI, TyG-WHtR, and TyG-WC with the risk of AHF across different subgroups. **Figure 6** presents the results of subgroup analyses for the association between the TyG-BMI and AHF. Our findings revealed a significantly stronger association between the TyG-BMI index and AHF only in the subgroup with the LAD artery as the culprit vessel (*P* < 0.05, *P* for interaction < 0.05). However, the relationship between TyG-BMI and AHF was not affected by age, Scr, UA, HDL-C, LDL-C, ApoB/A1, cardiac troponin I, gender, hypertension, diabetes mellitus, or the number of stents, with no statistically significant interaction observed between TyG-BMI and these variables (*P* for interaction > 0.05). **Supplementary Figures 7**–**9** detail the results of subgroup analyses for the associations of the TyG index, TyG-WC, and TyG-WHtR indices with AHF. No significant effects of subgroup variables on the associations between TyG-WC, TyG-WHtR, TyG-BMI and AHF were observed in any of these analyses (all *P* for interaction > 0.05). In conclusion, these findings indicate that the effects of TyG-related indices on AHF remain relatively stable across different levels of the aforementioned variables. This highlights their potential value in identifying and managing individuals at an increased risk of AHF within specific subgroups.

## Discussion

In this study, we analyzed in-hospital patients who had suffered AMI within the preceding 3 years, and comprehensively evaluated the associations of the TyG index and its related measures (TyG-WHtR, TyG-WC, TyG-BMI) with the occurrence of in-hospital AHF following emergency PCI in this population. The results showed that the levels of TyG, TyG-BMI, TyG-WHtR, and TyG-WC were significantly higher in patients with AHF than in those without AHF. In the multivariable Logistic regression model, each one-unit increase in the TyG index was associated with a 2.21-fold increased risk of AHF (OR: 2.21, 95% CI: 1.19–4.14, *P* < 0.01), while each one-unit increase in the TyG-WHtR index was linked to a 2.29-fold elevated risk (OR: 2.29, 95% CI: 1.35–3.91, *P* < 0.01). Further stratification of these indices by quartiles revealed that, across all four models, the higher quartiles of the TyG-WHtR exhibited the strongest association with an increased risk of AHF (OR: 2.29, 95% CI: 1.32–4.04, *P* < 0.01). ROC curve analysis demonstrated that, after adjustment for relevant confounding risk variables, the TyG-WHtR exhibited a significantly higher predictive value for the incidence of AHFA compared with other variables (AUC: 0.759, 95% CI: 0.721–0.797). Analyses using the RCS model demonstrated that the TyG index, TyG-BMI, TyG-WHtR, and TyG-WC remained linearly associated with AHF after adjustment for covariates (*P*-overall < 0.05, *P*-nonlinear > 0.05). Subgroup analyses identified a stronger association between TyG-BMI and AHF in individuals with the left anterior descending artery as the culprit vessel. Collectively, these findings highlight the important role of the TyG index and its derivatives that should be considered in assessing the risk of in-hospital AHF, particularly in AMI patients with left anterior descending artery occlusion.

The underlying mechanisms are primarily derived from previously published literature and remain speculative. The TyG index, as well as its combined indicators including the TyG-WHtR, TyG-WC, and TyG-BMI, are associated with the incidence of in-hospital AHF following PCI in patients with AMI. This correlation may be related to the synergistic pathogenic mechanisms underlying IR, metabolic disturbances, and obesity-associated injuries (19). The potential mechanisms are described as follows: (1) Following AMI, the body enters a stress state characterized by sympathetic activation, elevated inflammatory factor release, and increased glucocorticoid levels, all of which are linked to reduced insulin sensitivity in peripheral tissues. This reduction in insulin sensitivity may be accompanied by a shift in systemic energy supply toward lipid metabolism-a relatively inefficient process that may increase oxygen consumption. Such alterations can further exacerbate the imbalance between energy supply and demand in the ischemic myocardium, which may contribute to the pathogenesis of heart failure (20, 21); (2) IR is associated with inhibited nitric oxide (NO) synthesis and enhanced endothelin release, which may represent one cause of coronary microvascular endothelial dysfunction. Microcirculatory vessels already compromised by AMI may undergo vasospasm, leading to further hypoperfusion. This process can aggravate myocardial injury in ischemic regions and promote myocardial remodeling in viable myocardium (22, 23); (3) Inflammation and oxidative stress stimulate fibroblast proliferation and accelerate myocardial fibrosis, resulting in increased ventricular wall stiffness and impaired diastolic function. Concurrently, the coexistence of cardiomyocyte apoptosis and hypertrophy leads to thinning of the left ventricular wall, ventricular dilatation, and reduced ejection fraction, ultimately triggering the onset and progression of AHF (24–26).

Previous studies have demonstrated a J-shaped association between higher TyG-related indices and the risk of heart failure development (27), whereby the incidence of HF exhibits two phases of slow and rapid increase with the elevation of TyG-related indices. Additionally, these findings revealed no association between the TyG index and the risk of heart failure in the elderly population. In contrast to their findings, a study by Zhang F et al. confirmed a positive correlation between the TyG index and the risk of heart failure in the healthy population (28). Our study further confirmed a linear increase in the incidence of AHF with elevations in the TyG index and its combined indices (TyG-WHtR, TyG-WC, TyG-BMI) among patients with AMI. The discrepancies in study conclusions may be attributed to the coexistence of multiple comorbidities and physiological changes in the elderly population, as well as the fact that the study cohort in the present research comprised AMI patients with elevated blood glucose and lipid levels. This association may be attributed to “differences in obesity phenotypes.” Previous studies have shown that abdominal obesity (android fat distribution) is closely associated with adverse cardiometabolic risk and poorer prognosis of heart failure. In contrast, the gluteal obesity (gynoid fat distribution) may exert a protective effect, and its mechanism may be related to a more favorable adipokine profile and reduced visceral fat accumulation. These findings emphasize that fat distribution has an important impact on cardiovascular prognosis (29, 30). Notably, we observed a stronger association between TyG-BMI and AHF in patients with LAD coronary artery as the culprit vessel. This may be attributed to the fact that the LAD is one of the most critical vessels in coronary artery disease, and LAD lesions are frequently associated with a higher risk of myocardial ischemia and adverse prognosis. Furthermore, elevated BMI may contribute to increased volume overload. However, these subgroup findings should be regarded as exploratory observations and require further validation in independent cohorts or prospective studies (31, 32). Furthermore, a study by Nan Chen et al. demonstrated that among patients with ST-segment elevation myocardial infarction undergoing primary PCI, the incidence of early-onset HF was significantly higher in women than in men (29% vs. 14.8%) (33). Our study further validated this finding, with results showing a significantly higher incidence of AHF in women compared to men (22.3% vs. 16.0%). At the same time, our findings also indicated that TyG-WHtR had a stronger association with the risk of post-PCI AHF in AMI patients (OR: 2.29, 95% CI: 1.32–4.04, *P* < 0.01). This conclusion was consistent with the findings of a study by Xinyang Dui et al. (OR: 1.33, 95% CI: 1.01–1.75, *P* < 0.01), and the results revealed that TyG-WHtR, regardless of its high or low levels, was associated with an increased risk of HF (12). The discrepancy in OR may be attributed to the distinct study populations: our study included patients with AMI, whereas the comparator study focused on healthy elderly individuals. The AMI cohort in our research presented with a relatively higher degree of metabolic stress and potentially greater visceral fat accumulation, which may underpin the observed differences in OR values.

The findings of this study hold important implications for clinical practice and patient self-management. First, the TyG index and its combined indices (TyG-WHtR, TyG-WC, TyG-BMI) may serve as potential predictors of AHF following PCI in patients with AMI. Elevated TyG-WHtR levels are associated with an increased risk of AHF, suggesting that closer monitoring and early intervention may be warranted for these patients. Second, based on measurements of TyG-related indices, targeted health education can be provided to high-risk patients in clinical practice. Patients may be guided to reduce these indices through weight control, appropriate waist-to-height ratio management, and improved glucose and lipid metabolism. Such strategies may enhance patients’ awareness of postoperative health management and adherence to interventions, supporting the development of a patient-centered, comprehensive care model. Finally, measurement of TyG-related indices is convenient, cost-effective, and easy to perform without complex equipment. These indices may therefore be applicable in various medical institutions, especially primary care settings. Wider implementation could improve the accessibility of AHF risk stratification after PCI in AMI patients and facilitate early identification and intervention of postoperative cardiovascular complications. However, this study has several limitations. First, its retrospective design limits the ability to establish definitive causal relationships. Although multivariate adjustment and subgroup analyses were used to minimize confounding, several unmeasured factors may still have influenced clinical outcomes. These included infarct size, total ischemic time, and admission Killip class in patients with AMI, as well as specific phenotypic classification in patients with HF (e.g., acute left-sided versus right-sided HF) and the completeness of revascularization therapy. All of these factors are strongly associated with prognosis after PCI. Their exclusion from analytical models may have introduced bias into the observed associations, further restricting causal inference. Future studies with similar designs should collect more comprehensive baseline clinical data and systematically incorporate core metrics such as infarct size, ischemic duration, cardiac functional class, heart failure phenotype, and revascularization completeness. Such approaches would support more precise investigation of causal relationships between TyG-related indices and AHF following PCI. Second, this study primarily evaluated the baseline TyG index and its composite indices (TyG-WHtR, TyG-WC, TyG-BMI), but did not dynamically assess changes in insulin resistance during the clinical course. Third, categorization of some continuous variables into quartiles and use of ROC-derived cut-off values may have reduced statistical power and increased the risk of model overfitting. Moreover, multiple comparisons in subgroup and multivariable analyses could inflate Type I error rates and produce spurious positive associations. Subsequent studies should employ formal multiple testing corrections to improve statistical robustness. Finally, this study did not conduct internal or external validation, leading to uncertainties regarding the robustness and reproducibility of the observed associations. Future studies should implement internal validation strategies such as bootstrapping and cross-validation, or perform multi-center external validation, to improve the reliability of the conclusions.

## Conclusion

In conclusion, the findings of this retrospective study indicate that higher TyG index and its combined indices (TyG-WHtR, TyG-WC, TyG-BMI) are closely associated with the risk of AHF in patients with AMI following emergency PCI, among which TyG-WHtR exhibits the strongest correlation. These results suggest that TyG-related indices can serve as a valuable prognostic tool for AMI patients after emergency PCI, helping clinicians conduct early risk assessment and timely intervention to improve patients’ prognosis.

## Data Availability

The original contributions presented in the study are included in the article/**Supplementary Material**. Further inquiries can be directed to the corresponding author.
