# Supplementary material for: Association between TyG-related indices and in-hospital acute heart failure in patients with acute myocardial infarction after emergency percutaneous coronary intervention
Source: Front Endocrinol (Lausanne). 2026 Mar 6;17:1798955. doi: 10.3389/fendo.2026.1798955 (PMC13002376; doi:10.3389/fendo.2026.1798955)
Supplement: Supplementary file 1 [file DataSheet1.docx]

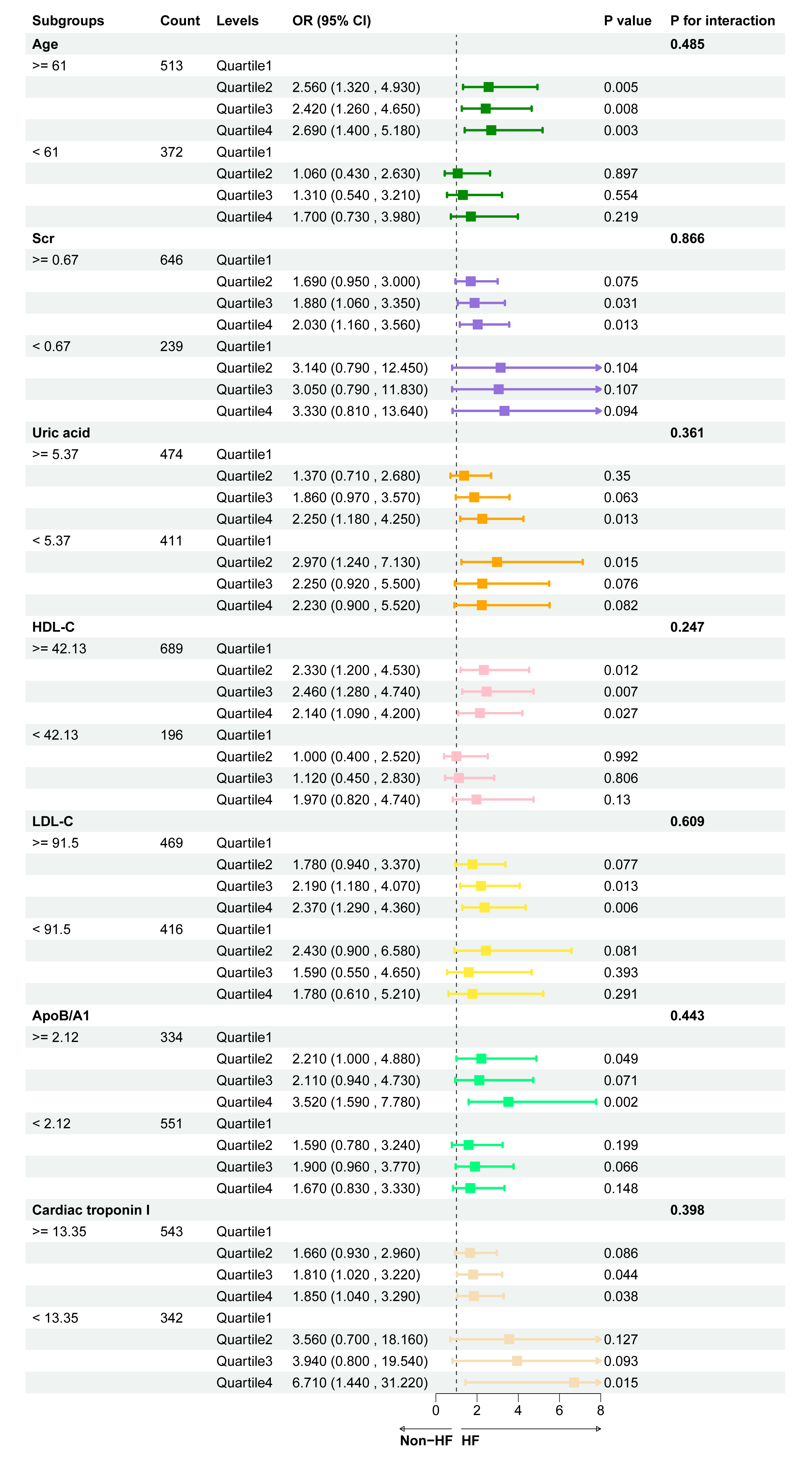


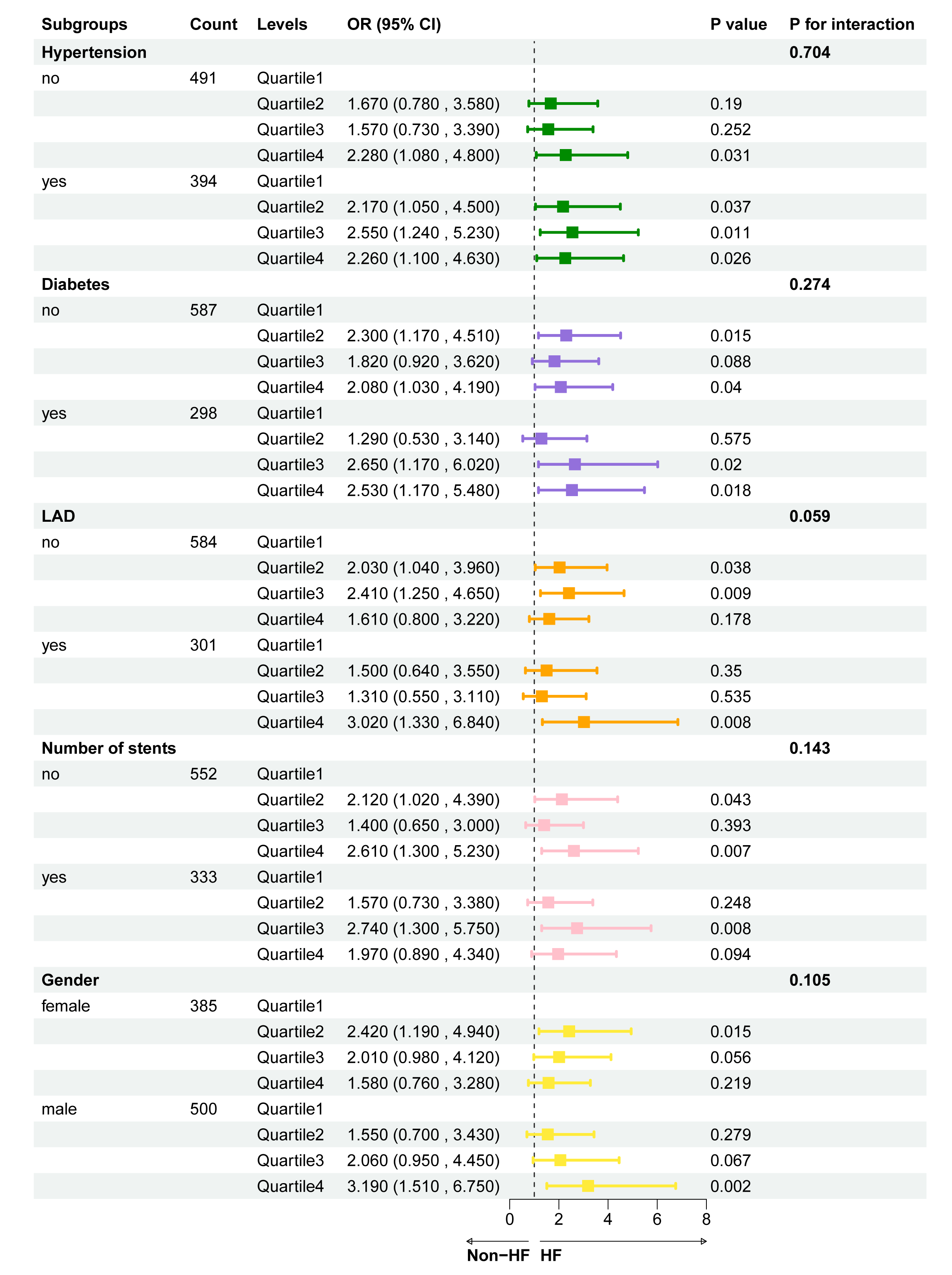


**Fig S7** The results of subgroup analyses for the associations of the TyG index with AHF


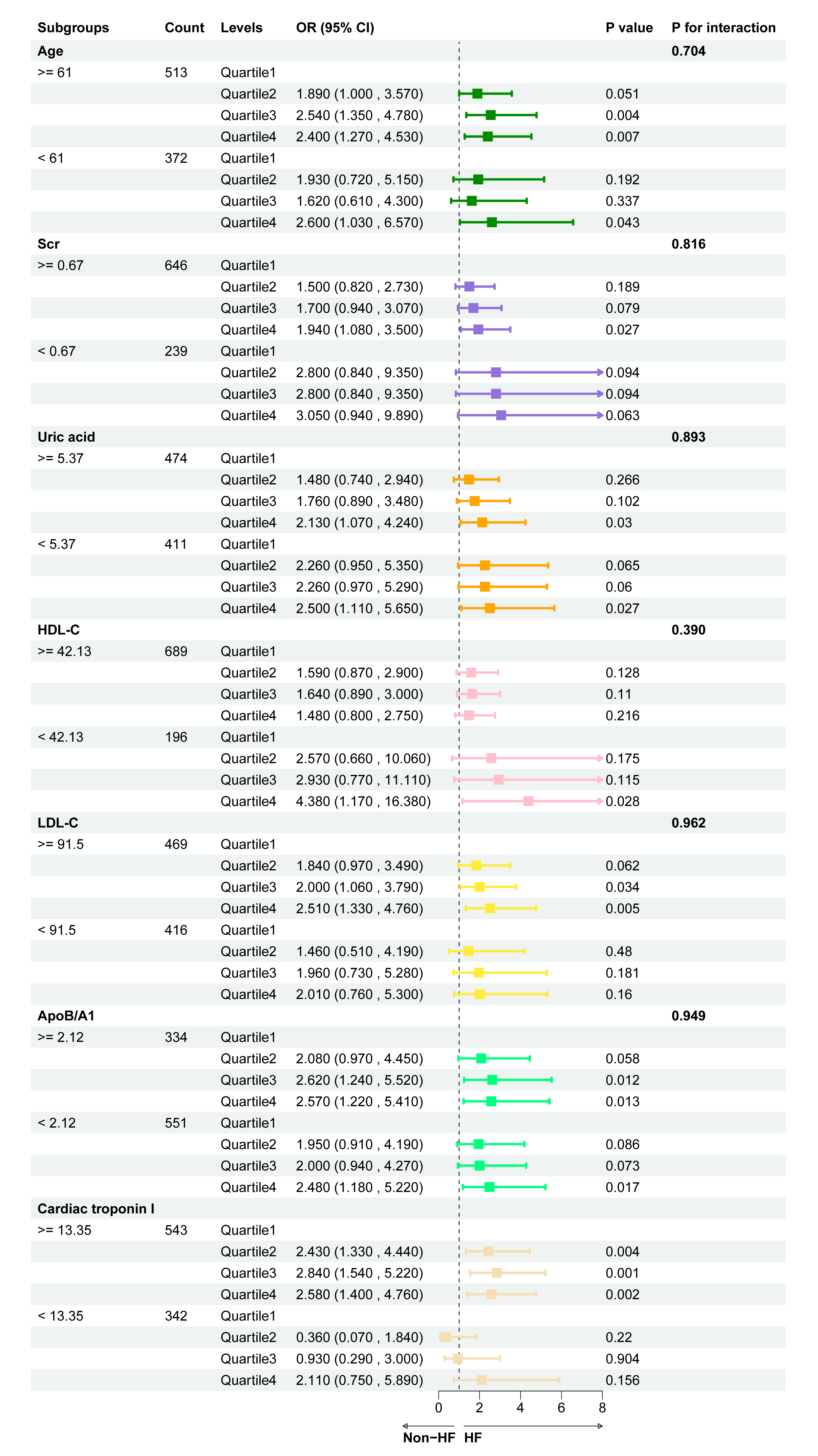


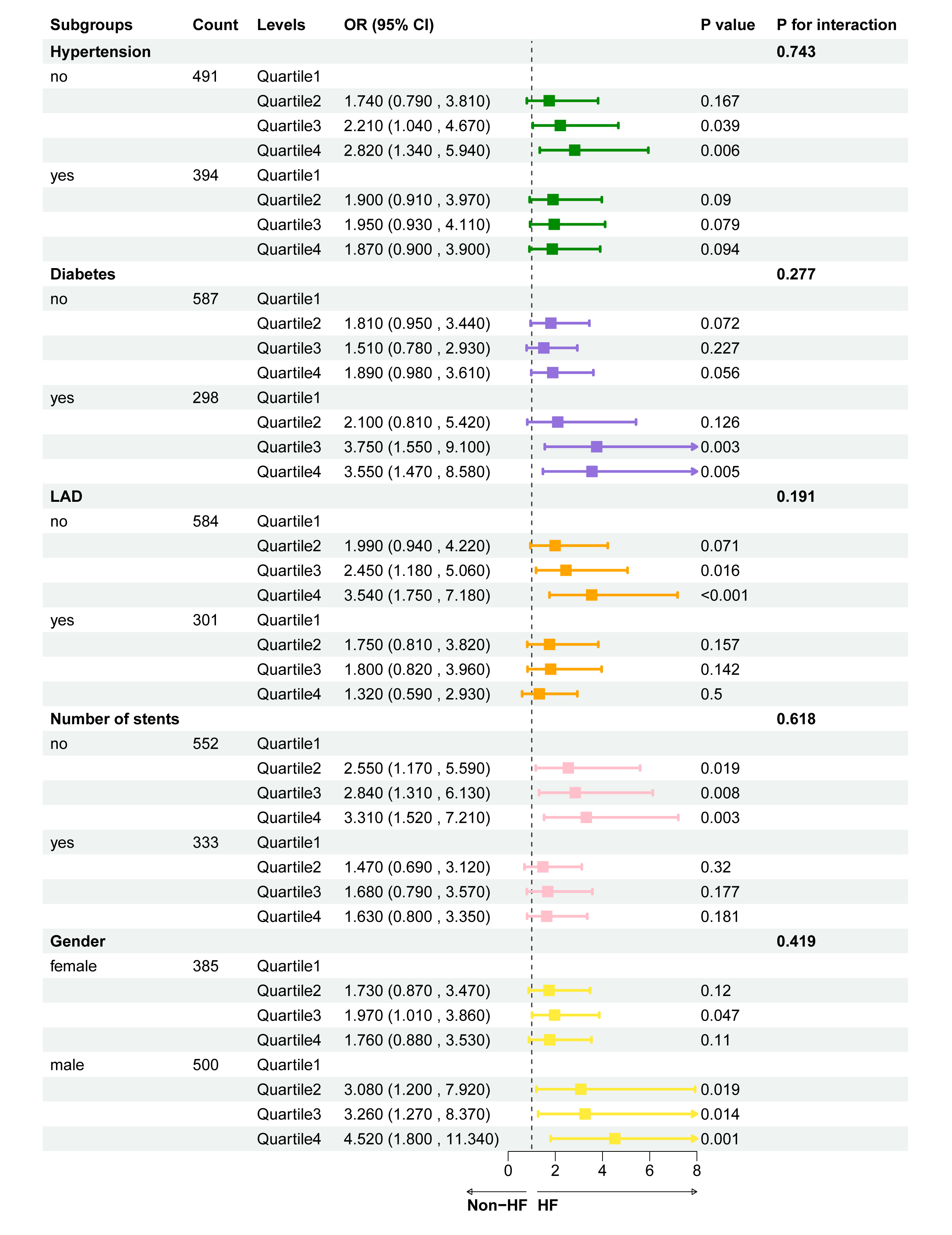


**Fig S8** The results of subgroup analyses for the associations of TyG-WC with AHF


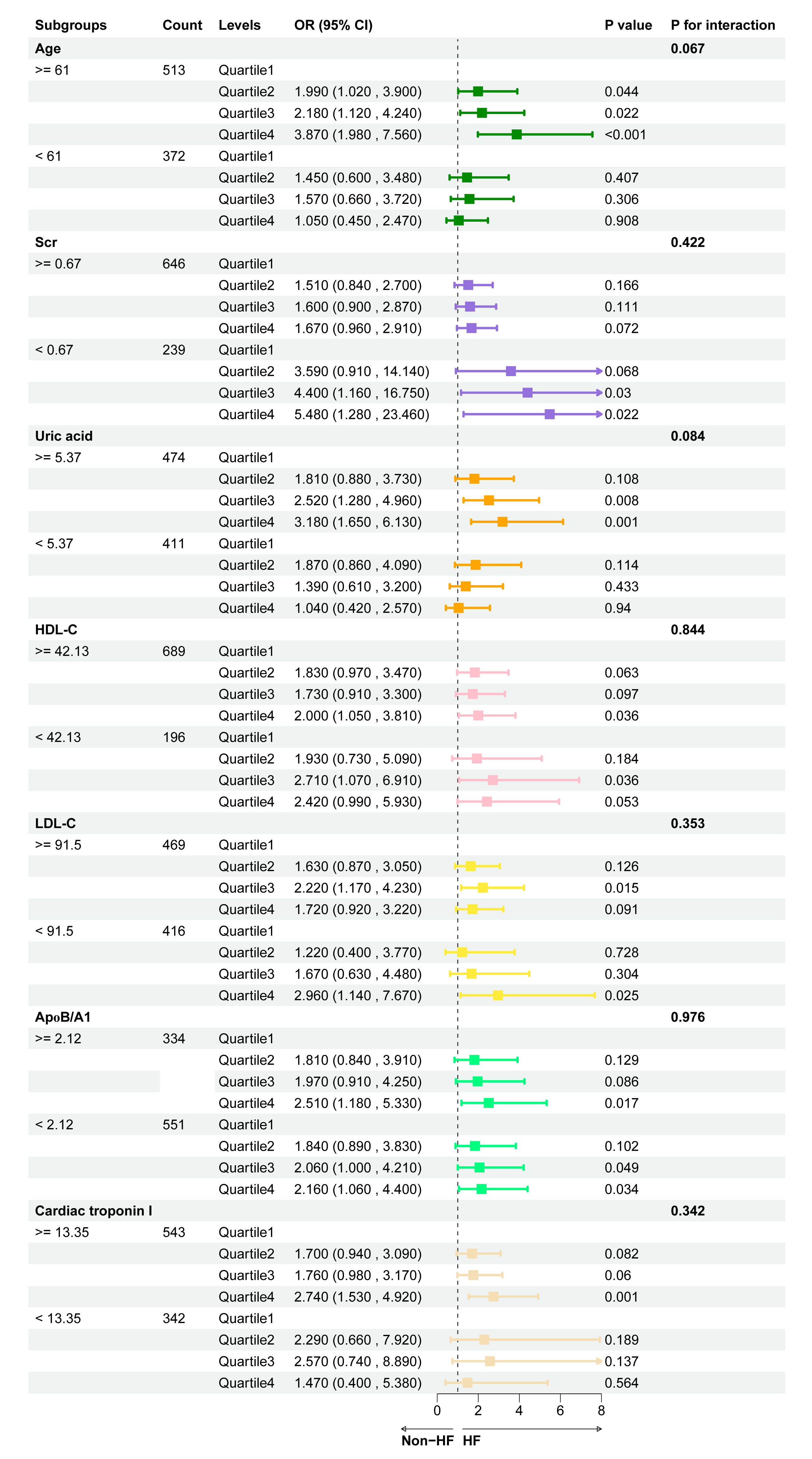


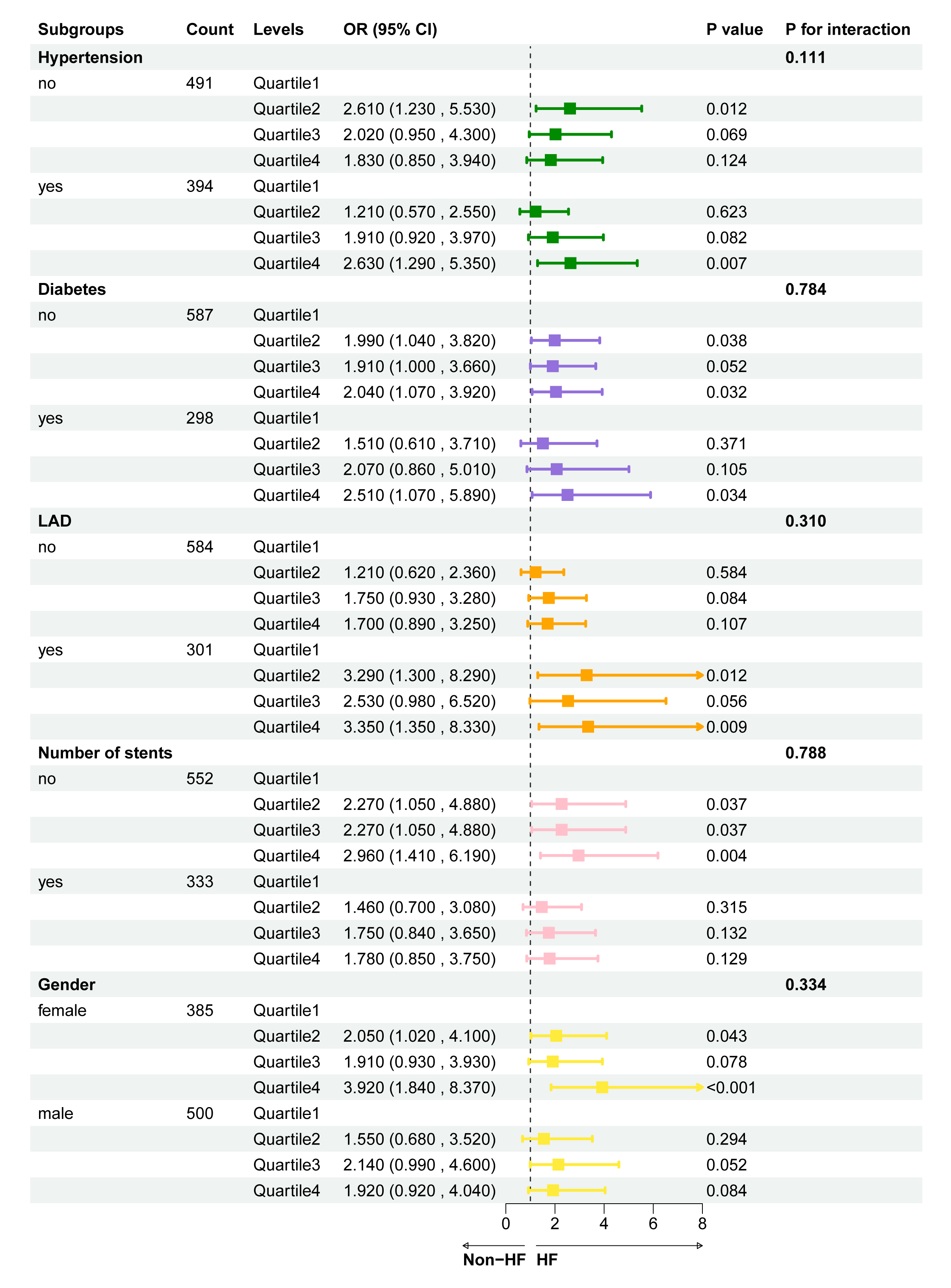


**Fig S9** The results of subgroup analyses for the associations of TyG-WHtR with AHF
